# The Transcriptomic Signature of Tigecycline in *Acinetobacter baumannii*

**DOI:** 10.3389/fmicb.2020.565438

**Published:** 2020-10-27

**Authors:** Liping Li, Karl A. Hassan, Sasha G. Tetu, Varsha Naidu, Alaska Pokhrel, Amy K. Cain, Ian T. Paulsen

**Affiliations:** ^1^Department of Molecular Sciences, Macquarie University, Sydney, NSW, Australia; ^2^School of Environmental and Life Sciences, University of Newcastle, Callaghan, NSW, Australia; ^3^ARC Centre of Excellence in Synthetic Biology, Macquarie University, Sydney, NSW, Australia

**Keywords:** tigecycline, antibiotic resistance, *Acinetobacter baumannii*, transcriptomics, bacterial physiological response to antibiotics

## Abstract

Tigecycline, a protein translation inhibitor, is a treatment of last resort for infections caused by the opportunistic multidrug resistance human pathogen *Acinetobacter baumannii*. However, strains resistant to tigecycline were reported not long after its clinical introduction. Translation inhibitor antibiotics perturb ribosome function and induce the reduction of (p)ppGpp, an alarmone involved in the stringent response that negatively modulates ribosome production. Through RNA sequencing, this study revealed a significant reduction in the transcription of genes in citric acid cycle and cell respiration, suggesting tigecycline inhibits or slows down bacterial growth. Our results indicated that the drug-induced reduction of (p)ppGpp level promoted the production but diminished the degradation of ribosomes, which mitigates the translational inhibition effect by tigecycline. The reduction of (p)ppGpp also led to a decrease of transcription coupled nucleotide excision repair which likely increases the chances of development of tigecycline resistant mutants. Increased expression of genes linked to horizontal gene transfer were also observed. The most upregulated gene, *rtcB*, involving in RNA repair, is either a direct tigecycline stress response or is in response to the transcription de-repression of a toxin-antitoxin system. The most down-regulated genes encode two β-lactamases, which is a possible by-product of tigecycline-induced reduction in transcription of genes associated with peptidoglycan biogenesis. This transcriptomics study provides a global genetic view of why *A. baumannii* is able to rapidly develop tigecycline resistance.

## Introduction

Tigecycline is a broad-spectrum antibiotic derived from minocycline and was the first glycylcycline class antibiotic approved for clinical use (Petersen et al., 1999). Compared to tetracyclines, tigecycline has increased antibacterial potency due to its higher binding affinity with the 70S ribosomes, or more specifically with the helix 31 and 34 of the16S rRNA on the head of the 30S subunit (Jenner et al., 2013). This effect inhibits the delivery of the thermo-unstable ternary complex elongation factor (EF-Tu)⋅GTP⋅aminoacyl-tRNA to the ribosomal A (aminoacyl) site and eventually perturbs polypeptide translation (Jenner et al., 2013). Additionally, tigecycline is not recognized by the major tetracycline resistance determinants, namely the major facilitator family (MFS) efflux pumps such as TetA/B which export tetracyclines out of the cell (Hirata et al., 2004), and ribosome protection proteins such as TetO and TetM which sequester tetracycline by binding to the tetracycline-stalled ribosome (Bergeron et al., 1996; Jenner et al., 2013).

Although tigecycline is not affected by common tetracycline resistance determinants, there have been increasing numbers of tigecycline resistant bacterial pathogens reported since its introduction in 2005. In Gram-negative organisms, the majority of such cases have been partially linked to the constitutive overexpression of resistance-nodulation-division (RND) efflux pumps, for instance MexXY-OprM in *Pseudomonas aeruginosa*, and AdeIJK and AdeABC in *Acinetobacter baumannii* (Dean et al., 2003; Visalli et al., 2003; Peleg et al., 2007; Damier-Piolle et al., 2008). Similarly, some Gram-positive organisms that display reduced susceptibility to tigecycline constitutively overexpress efflux pumps, such as the MATE family efflux pump MepA in *Staphylococcus aureus* (McAleese et al., 2005). Mutations of ribosomal protein genes and the 16S rRNA gene have also been shown to reduce tigecycline susceptibility of various organisms (Beabout et al., 2015; Lupien et al., 2015; Niebel et al., 2015), probably by affecting its target-site binding affinities. The proteobacterial TetX flavin-dependent monooxygenase, capable of inactivating tetracyclines, was also found to mediate tigecycline resistance when highly expressed (Moore et al., 2005). Plasmid- or mobile genetic element (MGE)-borne *tetX* genes have been identified in tigecycline resistant bacterial isolates from clinical and animal husbandry settings (He et al., 2019, 2020; Sun et al., 2019; Wang et al., 2019).

*Acinetobacter baumannii* is an opportunistic nosocomial human pathogen (Peleg et al., 2008). Tigecycline is one of the last resort therapies for the infections caused by carbapenem-resistant *Acinetobacter baumannii* strains, which have been listed as a top research priority for novel therapy development by the World Health Organization and the United States Centers for Disease Control and Prevention (CDC, 2019). Despite its broad spectrum antibacterial efficacy there have been reports of the development of resistant mutants during or after therapy and the United States Food and Drug Administration has warned of increased mortality risk for infections treated by tigecycline in comparison with other antibiotics (Navon-Venezia et al., 2007; Schafer et al., 2007; Anthony et al., 2008; Cai et al., 2011; Niu et al., 2019). The emergence of tigecycline resistant *A. baumannii* isolates can be correlated with a hypermutator phenotype (Hammerstrom et al., 2015). The plasmids or MGEs that carry *tetX* often also confer resistance to a wide range of other antibiotics (Morosini et al., 2006; Hornsey et al., 2011). Furthermore, the efflux pumps that can export tigecycline usually confer multidrug resistance (MDR) when overexpressed. Together these findings imply a future of tighter therapeutic options left for MDR *A. baumannii* infections or the emergence of pan-drug resistant infections. In this study, we sought to further characterize the genetic basis of tigecycline resistance in *A. baumannii*. To achieve this aim, RNA-Seq was used to analyse *A. baumannii* global transcriptomic response to tigecycline. We revealed that in addition to disrupting protein translation, tigecycline at a sub-inhibitory concentration also induced pleiotropic physiological effects, including differential expression of genes involved in RNA metabolism and DNA repair. These observations provide further insights into how *A. baumannii* may rapidly develop tigecycline resistance.

## Results and Discussion

A global clonal lineage I (GCI) *A. baumannii* 6772166 (Eijkelkamp et al., 2011; Hamidian and Hall, 2014) is an intermediate tigecycline resistant clinical isolate, with a minimum inhibitory concentration (MIC) between 2.5 and 5 μg/ml. When exposed to antimicrobials at a sub-inhibitory concentration, bacteria tend to evolve and develop antimicrobial resistance (Baym et al., 2016). In this study, we aimed to capture the physiological response to tigecycline in parental cells, rather than in the offspring population, which may be mixed by cells that have adapted to the drug. Because the *A. baumannii* doubling time at log phase is around 25 min and tigecycline slows down bacterial growth, this strain at mid-exponential growth phase was exposed to tigecycline at 2.5 μg/ml for 30 min, and the global transcriptomic response was analyzed via RNA-Seq.

**FIGURE 1:**
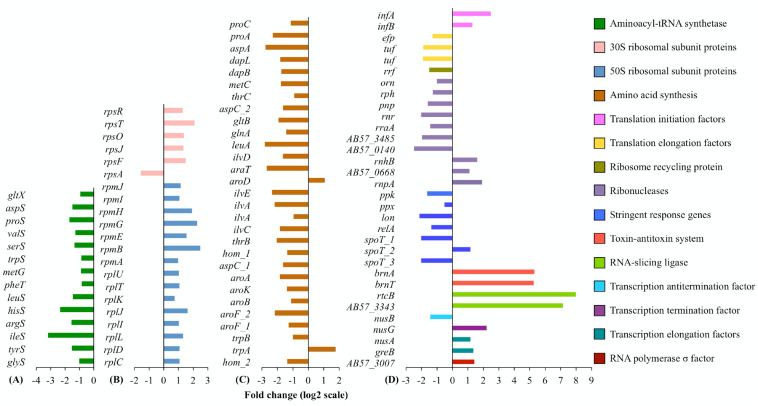
**(A)** Aminoacyl-tRNA synthetases, **(B)** ribosomal proteins, **(C)** amino acid synthesis, and **(D)** others. Differential expression of genes involved in, or affecting, protein translation. For each functional group or cellular pathway, only differentially transcribed genes with absolute fold changes >1 (log_2_ scale) and padj < 0.01 are shown. The bar charts are grouped by colors according to the biological functions of the genes assigned, with the color scheme on the right. Full information on gene function annotations is given in Supplementary Data.

Hua et al. has published similar work in a different *A. baumannii* clinical isolate, and their data revealed that tigecycline induces pleiotropic physiological impacts (Hua et al., 2014). Although not discussed in their paper, the data from Hua et al. (2014) also showed upregulation of genes encoding ribosomal proteins and drug efflux pumps, and downregulation of the genes involved in citric acid cycle and cell respiration chain. However, our current study provides additional physiological and biomolecular insights. We showed more than 1000 genes with a greater than two-fold change in gene expression following tigecycline exposure in *A. baumannii* 6772166 (Supplementary Figure 2 and Supplementary Data), indicating that tigecycline induces broad physiological changes. This widespread alteration in gene transcription may be partially explained by the significant expression changes in 76 genes encoding putative or characterized transcriptional regulators, the up regulation of one highly expressed σ^70^ factor homolog gene, and the altered expression of various ribonuclease genes (Figure 1D).

### Tigecycline Slows Down Protein Translation but Boosts Ribosome Synthesis

In line with the known mode of action of tigecycline, where its interaction with the 16S rRNA directly blocks the aminoacyl-tRNA from entering the ribosomal A site (Jenner et al., 2013), the RNA-Seq data shows clear evidence of ribosomal stalling after translational initiation. Fourteen genes encoding aminoacyl-tRNA synthetases (Figure 1A) and 27 genes responsible for amino acid synthesis (Figure 1C) were transcriptionally down regulated, possibly because of a reduced turnover of aminoacyl-tRNA/tRNA, and a reduced rate of amino acid incorporation into polypeptide chains. Correspondingly, transcript abundance was also decreased for two EF-Tu (*tuf*) genes and the EF-P encoding gene (EF-P, *efp*), which participates in peptidyl transferase activity on the ribosomal P (peptidyl) site (Figure 1D). Transcription of the ribosome recycling factor (RRF) encoding gene *frr* was also decreased in response to tigecycline (Figure 1D). RRF is required for 70S ribosome separation at the end of each peptide translation cycle and ribosomal subunit recycling for the subsequent round of translation (Wilson, 2009). These observations suggest a slower protein translation rate, and are consistent with the known initial antibacterial action of tigecycline in protein translation perturbation (Jenner et al., 2013). Decreased transcript levels of genes involved in amino acid synthesis and tRNA aminoacylation were also reported in *Streptococcus pneumoniae* when treated with translational inhibitors including tetracycline, chloramphenicol, erythromycin and puromycin (Ng et al., 2002). Our RNA-seq data also showed decreased transcriptional levels of twelve genes encoding proteases, potentially indicative of a slower protein degradation rate following tigecycline treatment.

The transcription of genes for sixteen 50S and five 30S ribosomal proteins were increased (Figure 1B), consistent with previous findings in *S. pneumoniae*, *Escherichia coli*, and *Haemophilus influenzae* following treatment with translational inhibitors including tetracycline and transcriptomic or proteomic measurement of expression changes (VanBogelen and Neidhardt, 1990; Evers et al., 2001; Ng et al., 2002). The *H. influenzae* study demonstrated that translational inhibitors increased the total RNA synthesis rate, and hence the corresponding rate of rRNA synthesis, as this is the major RNA species in the cell (Evers et al., 2001). rRNA synthesis is the rate-limiting step in ribosome synthesis and assembly (Maaløe and Kjeldgaard, 1966). Our study used rRNA-depleted samples, so we could not directly verify whether the rRNAs were increased in abundance upon tigecycline treatment. However, transcription was observed to increase for two translation initiation factors IF-1 (*infA*) and IF-2 (*infB*), required for the assembly of ribosome subunits to start a new protein translation cycle, consistent with the *A. baumannii* cellular response including synthesis of ribosomal proteins and rRNA and a greater need for ribosomal assembly.

The transcriptional changes in genes responsible for several cellular activities involved with guanosine tetraphosphate and guanosine pentaphosphate (p)ppGpp further support the notion that, like other translational inhibitor antibiotics, tigecycline could boost rRNA and ribosome biosynthesis in *A. baumannii*. (p)ppGpp is a negative effector of the *rrnB P1* promoter of rRNA operons (Murray et al., 2003). In adapting to amino acid starvation, overproduction of (p)ppGpp inhibits ribosome synthesis directly and mediates ribosomal protein degradation through Lon protease, via the stringent response (Kuroda et al., 2001; Wendrich et al., 2002). Upon tigecycline treatment, we observed decreased expression levels of various genes responsible for (p)ppGpp synthesis and degradation (Figure 1D, stringent response genes). These include *relA*, encoding a (p)ppGpp synthetase, *spoT*, encoding a synthetase and hydrolase of (p)ppGpp, and *lon*, encoding Lon protease. In addition, we observed decreased expression of genes impacting cellular levels of polyphosphate (polyP) – a known cofactor of Lon-mediated free ribosomal protein degradation (Kuroda et al., 2001) – including *ppk*, encoding polyphosphate kinase, and *ppx*, encoding exopolyphosphatase. These results, together with the observation of reduced (p)ppGpp cellular level in translational inhibitor antibiotics stressed bacterial cells (Evers et al., 2001), suggest that tigecycline could also induce the reduction of (p)ppGpp production in *A. baumannii*. Such reduced (p)ppGpp production would be expected to stimulate rRNA synthesis and inhibit Lon-mediated ribosomal protein degradation.

### Central Metabolism and Cell Division

**FIGURE 2:**
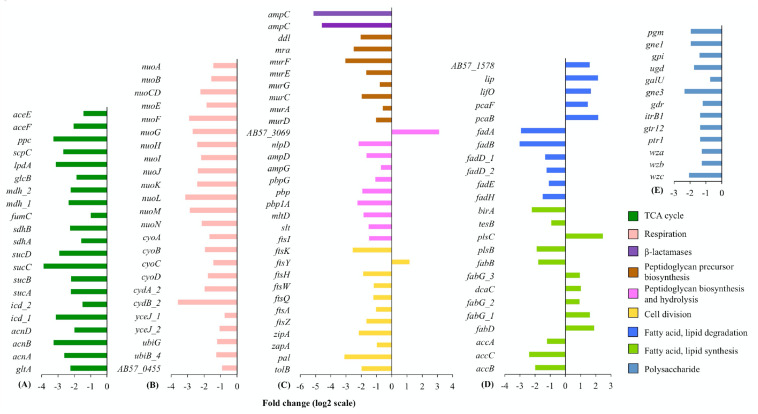
**(A)** TCA cycle, **(B)** respiration, **(C)** cell wall and cell division, **(D)** fatty acid and lipid, and **(E)** polysaccharide. Differential transcription of genes in key metabolic pathways. For each cellular pathway, only differentially transcribed genes with absolute fold changes >1 (log_2_ scale) and padj < 0.01 are shown. The bar charts are grouped by colors according to the biological functions of the genes assigned, with the color scheme on under panel **(E)**. Full information on gene function annotations is given in Supplementary Data.

Bacteriostatic antibiotics capable of inducing translation perturbation have been reported to have profound downstream consequences on suppressing bacterial metabolism, including the accumulation of metabolites from central metabolic and cellular respiration pathways (Lin et al., 2014; Lobritz et al., 2015). Our RNA-Seq data showed consistent tigecycline-induced reductions in gene expression for enzymes in the tricarboxylic acid (TCA) cycle and cellular respiration (Figures 2A,B). Transcription of the genes responsible for cell wall metabolism and cell division was also down regulated by tigecycline (Figure 2C). The effect of tigecycline on transcription of genes involved in translation perturbation, RNA degradation, TCA cycle and respiration, cell division and cell-wall synthesis, reflect the reduction in cellular growth rate in *A. baumannii* 6772166, potentially linked to tigecycline’s bacteriostatic effect (Cocozaki et al., 2016).

### Genes With Highest Transcriptional Increases Are Involved in RNA Metabolism

A plasmid-borne toxin-antitoxin (TA) system, homologous to a chromosomal TA system *brnT/brnA* first characterized in *Brucella abortus* (Heaton et al., 2012), showed 39-fold increased expression in response to tigecycline (Figure 1D). BrnT is a type II ribonuclease toxin which when co-expressed with the antitoxin protein BrnA forms a tetramer BrnT_2_-BrnA_2_ that neutralizes BrnT toxicity and functions as a strong autorepressor of its own operon (Heaton et al., 2012). However, a decrease in the cellular level of BrnA can de-repress expression of the TA system and lead to increased levels of BrnT, which in turn inhibits cell growth through RNA degradation, thus interfering with protein translation (Heaton et al., 2012). Chloramphenicol was shown to stimulate the overexpression of *brnT/brnA* in *B. abortus* (Heaton et al., 2012). A potential mechanism behind the increased transcription of this TA system in our current study is that the antitoxin is less stable and upon translational inhibition induced by either chloramphenicol or tigecycline, the antitoxin will become inactive, the TA complex will dissociate and thus transcription of the TA operon will be derepressed.

The two genes with the highest fold expression increase (252-fold) were *rtcB*, encoding a tRNA repair enzyme, and a hypothetical gene AB57_3343 directly downstream of *rtcB* (Figure 1D). *rtcB* was initially characterized in *E. coli* as a stand-alone ligase involved in healing and sealing broken tRNA-like stem-loop structures *in vitro*, and was later demonstrated to catalyze tRNA and mRNA repair in yeast *in vivo* (Tanaka and Shuman, 2011; Tanaka et al., 2011). We speculate that *rtcB* overexpression could be linked to *brnT/brnA* overexpression, as it may play a role evading the potential programmed RNA breakage mediated by this TA system, and RtcB could potentially serve as an indirect tigecycline induced stress-response determinant. Another possibility is that blockage of tRNAs to the ribosome caused by tigecycline leads to a cellular response in tRNA repair. *rtcB* was also significantly up-regulated in the previous study of *A. baumannii* MDR-ZJ06 transcriptomic response to tigecycline (Hua et al., 2014). In either case, given the very strong upregulation of transcription of this gene, RtcB could potentially serve as an indirect tigecycline induced stress-response determinant.

### Impacts on DNA Repair and Horizontal Gene Transfer

**FIGURE 3:**
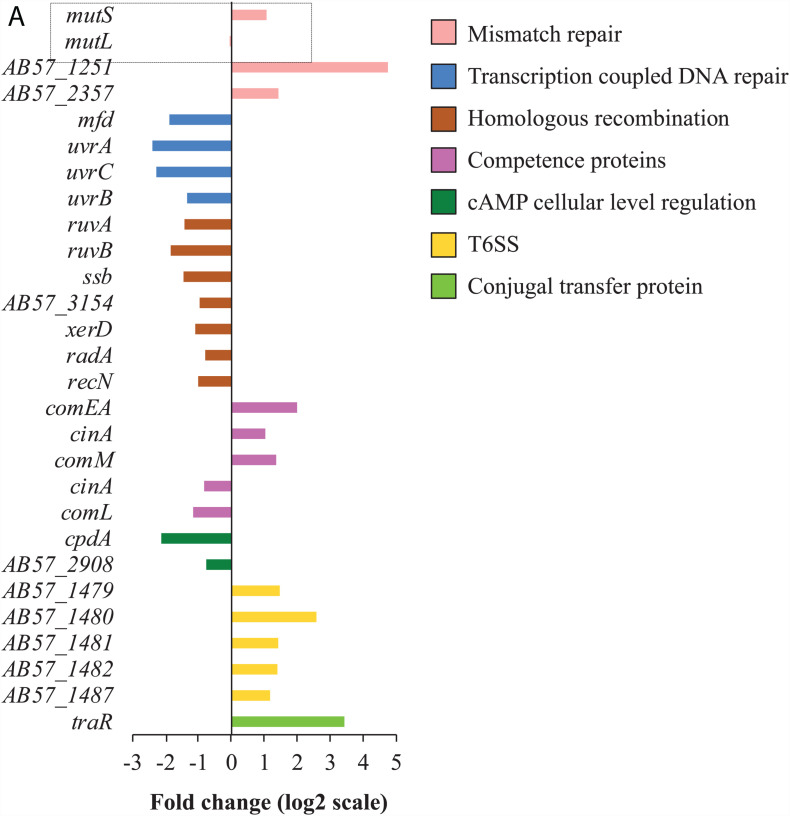
Tigecycline affects transcription of genes involved in DNA repair and HGT. **(A)** For each functional group, only differentially transcribed genes with absolute fold changes >1 (log_2_ scale) and padj < 0.01 are shown. The bar charts are grouped by colors according to the biological functions of the genes assigned, with the color scheme on the right. Full information on gene function annotations is given in Supplementary Data.

Transcriptomic data presented here suggests that tigecycline may impact DNA repair and DNA competence and mobility. For instance, the transcription-coupled nucleotide excision repair (TCR) genes *mfd*, *uvrA*, *uvrB*, and *uvrC* all showed decreased transcription (Figure 3). Mfd and UvrA are important in bacterial mutagenesis and evolution of antimicrobial resistance during host infection where DNA replication in the bacterial cells is low while transcription is still active (Ragheb et al., 2019). Furthermore, the expression of the transcription EF gene *greB* that interferes TCR activities was increased, and the expression of (p)ppGpp synthesis genes were decreased, which are required for efficient TCR activities (Figure 1D; Epshtein et al., 2014; Kamarthapu et al., 2016). These findings, that tigecycline exposure diminishes TCR activities via mechanisms including (p)ppGpp reduction, collectively suggest that such exposure can result in an increased TCR-related mutation rate.

Increased expression was observed for *mutS*, encoding the DNA mismatch repair (MMR) enzyme, and two other putative DNA repair genes AB57_2357 and AB57_1251 (Figure 3). Tigecycline has been reported to generate tigecycline-resistant hypermutator strains of *A. baumannii* either *in vitro* (in a bioreactor) or *in vivo* (patient with tigecycline therapy), linked to insertion sequence (IS)-mediated inactivation of *mutS* (Hammerstrom et al., 2015). Speculatively, the emergence of tigecycline resistant strains linked to insertional disruption of the *mutS* gene, may reflect that *mutS* is the principle mis-match repair system induced by tigecycline at sub-inhibitory concentrations (Figure 3); and thus inactivation of *mutS* would substantially enhance mutation rates.

Tigecycline treatment increased expression of various genes associated with DNA mobility, including twenty-five putative transposase genes and four homologs of the DNA mobilization gene *bmgB* (Supplementary Figure 4), which could be a potential factor in the rapid emergence of IS-inactivated *mutS* mutants in *A. baumannii* previously reported (Hammerstrom et al., 2015). Genes involved in DNA uptake were also induced by tigecycline treatment including the cell competence genes *comM*, *cinA*, and *comEA*, and conjugal transfer protein *traR* genes. These changes in expression of DNA competence and mobilization genes suggests that tigecycline may affect horizontal gene transfer (HGT) rates. Consistent with this hypothesis, an operon encoding the type VI secretion system (T6SS) showed increased expression following tigecycline treatment (Figure 3). The T6SS in *Vibrio cholerae* was shown to be up-regulated through competence induction, releasing the DNA of the surrounding non-immune cells through deliberate killing to make the DNA available for uptake (Borgeaud et al., 2015). Expression of a lytic transglycosylase gene, proposed to play a role in creating space within the peptidoglycan sacculus for the insertion of cell-envelope spanning structures, such as T6SS, was also induced by tigecycline (Scheurwater et al., 2008).

### Tigecycline and β-Lactams

The most highly down-regulated genes in this study were two *ampC*β-lactamase-encoding genes (Figure 2C), whose transcription was reduced by 35-fold and 24-fold. AmpC β-lactamases are active against five classes of β-lactams (Jacoby, 2009). β-lactams can induce a toxic malfunctioning of the cycle of peptidoglycan synthesis, hydrolysis and recycling, and induction of β-lactamase genes typically correlates with peptidoglycan recycling (Jacoby, 2009; Li et al., 2016b). Interestingly, tigecycline also induced decreased transcription of four DD-carboxypeptidase-encoding genes (Supplementary Data). Speculatively, reduced expression of peptidoglycan biosynthesis genes (Figure 2C) might lead to, or be accompanied by, reduced production of N-acetylglucosamine moieties, which positively affect β-lactamase gene expression (Jacobs et al., 1994, 1997). This supports an intriguing possibility that tigecycline and β-lactams could be used synergistically in combination therapy, as has been hypothesized previously (Tumbarello et al., 2012). To investigate the potential synergy between tigecycline and β-lactams, we conducted checkerboard assays on *A. baumannii* 6772166 with tigecycline and five AmpC β-lactam substrates, respectively, including piperacillin, carbenicillin, ceftriaxone, cefotaxime and cefuroxime (Garcia, 2010). While the fractional inhibitory concentration indexes indicated no clinically significant synergy, inclusion of tigecycline at 2.5 μg/ml resulted in a four to eight-fold decrease in the MIC of these five β-lactams for *A. baumannii* 6772166 (Table 1). Bactericidal antibiotics, such as β-lactams, induce reactive oxygen species (ROS) dependent killing (Kohanski et al., 2007); whereas tigecycline at a subinhibitory concentration causes the downregulation of TCA cycle and respiration, both of which would likely limit ROS generation (Lobritz et al., 2015). This may explain why we only observed low synergy between tigecycline and the β-lactams, despite tigecycline-induced *ampC* downregulation.

**TABLE 1:** Results of checkerboard assay between tigecycline and β-lactams^a^

	β-lactam MIC (mg/ml)^*b*^		Tigecycline MIC (μg/ml)^*b*^		FICI^*c*^
	Alone	With tigecycline	Alone	With β-lactam	
Piperacillin	2	0.25	5	2.5	0.625
Carbenicillin	8	2	5	2.5	0.75
Ceftriaxone	2	0.5	5	2.5	0.625
Cefotaxime	0.5	0.125	5	2.5	0.75
Cefuroxime	2	0.5	5	2.5	0.75

*^*a*^For each of the five checkerboard assays, two biological replicates were performed. ^*b*^The wells picked for FICI calculation were with lowest growth inhibitory concentration of β-lactams. This was to show that tigecycline at subinhibitory concentration increased *A. baumannii* susceptibility to β-lactams. The wells with similar FICI but of lower tigecycline inhibitory concentrations were not shown. ^*c*^The defined clinically significant synergy is of FICI ≤ 0.5.(Odds, 2003).*

### Drug Efflux and Uptake

**FIGURE 4:**
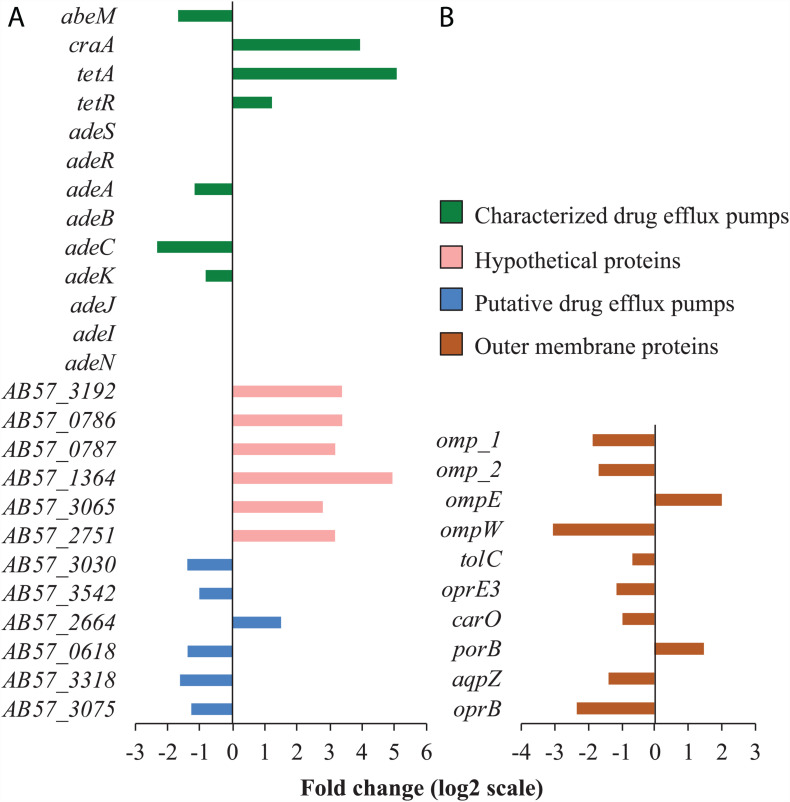
Tigecycline impact on the transcription of genes involved in membrane permeability and transport. **(A)** The characterized drug efflux pumps presented here are either the ones differentially regulated, or the ones known in conferring tigecycline resistance. The hypothetical proteins are responsive to tigecycline and predicted to have transmembrane helixes. **(B)** Only the differentially regulated OMPs are presented. All the genes on the panel **(A,B)** have absolute fold change more than 1 (log_2_ scale) with statistical significance (padj < 0.01). Full information on gene function annotations is given in Supplementary Data.

A common mechanism for resistance to tetracycline antibiotics involves reducing drug intracellular concentration via efflux pumps and/or down regulation of the outer membrane proteins (OMP) responsible for drug uptake (Piddock, 2006). The multidrug efflux pumps previously characterized in *A. baumannii* associated with tigecycline resistance are AdeABC and AdeIJK (Peleg et al., 2007; Damier-Piolle et al., 2008). The AdeABC-mediated tigecycline resistant *A. baumannii* strains reported previously showed mutations in either *adeR* or *adeS*, leading to its constitutive overexpression and hence tigecycline resistance (Peleg et al., 2007; Sun et al., 2014). However, in our RNA-Seq data *adeA* and *adeC* were down-regulated by tigecycline by more than two-fold, and the *adeIJK* operon was not differentially transcribed (Figure 4A). Although conferring resistance to tigecycline, the expression of *adeABC* and *adeIJK* are not positively responsive to the drug. This probably explains why constitutive overexpression of these two RND systems is commonly observed in tigecycline resistant *A. baumannii* strains.

Two genes encoding characterized drug efflux pumps (*tetA* and *craA*) and ten genes encoding hypothetical proteins with predicted transmembrane helices were found to be transcriptionally responsive to tigecycline (Figure 3A). The tetracycline exporter gene *tetA* and its transcriptional repressor gene *tetR* were both upregulated (Figure 3A), which is consistent with previous observations that tigecycline could induce *tetA* overexpression in *E. coli* but *tetA* does not confer tigecycline resistance (Hirata et al., 2004). CraA is a MDR efflux pump that transports chloramphenicol (Roca et al., 2009) and other antimicrobials (Li et al., 2016a). To further investigate whether CraA and the ten hypothetical membrane proteins do confer tigecycline resistance, these genes were heterologously overexpressed in *E. coli*. However, no significant resistance phenotype was observed for any of these clones, suggesting that although transcriptionally responsive to tigecycline, they do not mediate tigecycline resistance, or at least not at an easily detectable level.

Tigecycline treatment also led to decreased transcription of several OMP genes (Figure 3B) and genes involved in capsular polysaccharide biosynthesis and export (Figure 2E). These changes might affect bacterial outer membrane permeability and thus tigecycline uptake.

## Conclusion

In this study, we used RNA-Seq to show that exposure to a sub-inhibitory concentration of tigecycline in *A. baumannii* affected transcription of genes involved in protein translation as expected, but also resulted in significant gene transcriptional changes associated with RNA metabolism, DNA mismatch repair, HGT and genetic element mobilization. It also reduced the transcriptional levels of genes involved in the biogenesis of key cellular components, including the cell membrane, peptidoglycan, respiration chain and TCA cycle. Tigecycline was found to increase the susceptibility of *A. baumannii* to five β-lactams, which is hypothesized to be correlated with the tigecycline-induced reduction of peptidoglycan biogenesis accompanied by the downregulation of two β-lactamase encoding genes.

The core tigecycline cellular response appears to be mainly coordinated through down-regulation of (p)ppGpp, which modulates ribosome production to counteract translational inhibition effect of tigecycline, and impairs TCR-related DNA mismatch repair. Furthermore, tigecycline may increase rates of IS element transposition. These effects collectively may increase the rate of emergence of tigecycline-resistant mutants. One question arising from this study is whether the other translation-inhibiting antibiotics affect DNA repair pathways in the same way as seen here for tigecycline. Ribosome stalling and (p)ppGpp reduction are common translational inhibitor-induced responses in various bacterial species, therefore the related physiological changes, such as TCR-related DNA mismatch repair, are likely the core tigecycline transcriptomic signatures shared across many bacteria. Given the known variation in genotypes and phenotypes across characterized *A. baumannii* isolates, it is likely there will be some degree of strain to strain variation in transcriptional responses to tigecycline.

Tigecycline, a treatment of last resort for infections by opportunistic, MDR pathogens, remains an important clinical resource. Here we show that *A. baumannii* mounts a more complex suite of cellular responses to such treatment than previously appreciated and reveal why hypermutation can occur during treatment.

## Materials and Methods

### Bacterial Strains and Genome Sequences

*Acinetobacter baumannii* 6772166, kindly gifted by Dr. Melisa Brown (Eijkelkamp et al., 2011), with a tigecycline MIC between 2.5 and 5 μg/ml, was used for this transcriptomic study. The genomic DNA (gDNA) was sequenced (GEO accession no: GSE131451) and shown to share high identity to the genome sequence of AB0057 (Supplementary Figure 1). The plasmid and chromosome sequences of *A. baumannii* AB0057 were used as the reference sequence for the subsequent RNA-seq read alignment and statistical analyses. *E. coli* strain DH5α (α-select chemically competent cells, Bioline Australia) was used for heterologous overexpression and functional characterization for the putative transport systems.

### Tigecycline Transcriptomic Analysis by RNA-Seq

*Acinetobacter baumannii* 6772166 Muller-Hinton (Oxoid, United States) broth cultures at OD_600_ 0.6 with and without 2.5 μg/ml tigecycline (Sigma-Aldrich, United States) treatment were grown for 30 min at 37°C with shaking. Three biological replicates were prepared. The total RNA of each sample was extracted using the miRNeasy Mini Kit (QIAGEN, Germany) following the manufacturer’s instructions. rRNA was depleted by using the Ribo-Zero Magnetic kit (bacteria) (Illumina, Inc., United States), and converted to cDNA which was sequenced via Illumina HiSeq2500 at Ramaciotti Center for Genomics. Nearly 75 million unique 101 bp reads were obtained from each RNA sample representing more than 310-fold genome coverage. The sequence reads from the 6 RNA-Seq samples (GEO accession no: GSE131451) were aligned against the *A. baumannii* AB0057 genome and transcriptional coverage for each gene was determined using EDGE-pro (Estimated Degree of Gene Expression in Prokaryotic Genomes) (Magoc et al., 2013). More than 99% of the RNA-Seq reads of each sample were mapped to AB0057 genome sequence. Genes with significantly different transcription between the control and experimental samples were identified using the DEseq R package (Supplementary Data). Gene differential expression was visualized by metabolic cellular view in Biocyc (Caspi et al., 2016; Karp et al., 2019).

### Functional Validation of Membrane Transporters Through Heterologous Overexpression

Eight genes, including two efflux pumps and six hypothetical proteins with at least three transmembrane helices predicted by the TMHMM Server v.2.0., were cloned into pTTQ18 and overexpressed in *E. coli* DH5α as previously described (Hassan et al., 2011). The MIC of each *E. coli* clone was determined through two-fold serial broth dilution in MH broth according to the guidelines of CLSI (Clinical and Laboratory Standards Institute), with two biological replicates.

### Tigecycline and β-Lactam Antibiotics Synergism Validation Through Checkerboard Assay

The synergism between tigecycline and five β-lactam antibiotics, namely piperacillin, carbenicillin, ceftriaxone, cefotaxime and cefuroxime (all from Sigma-Aldrich, United States) were determined through broth microdilution checkerboard assay following previously developed methodology (Garcia, 2010). Two independent biological repeats were performed for each combination set.

## Data Availability Statement

The datasets presented in this study can be found in online repositories. The names of the repository/repositories and accession number(s) can be found in the article/Supplementary Material.

## Author Contributions

IP, KH, and LL conceptualized the work. LL conducted all experiments with assistance from AP and VN. LL performed transcriptomic the data analyses with significant assistance and contribution from ST. LL, IP, KH, AC, and ST worte the manuscript. All authors contributed to the article and approved the submitted version.

## Conflict of Interest

The authors declare that the research was conducted in the absence of any commercial or financial relationships that could be construed as a potential conflict of interest.

## Supplementary Material

Supplementary Figure 1 Mauve visualization of genomic identity between *A. baumannii* AB0057 and *A. baumannii* 6772166. The horizontal track on the top represents the chromosome sequence of *A. baumannii* AB0057, and the one on the bottom represents the rearranged contig sequences of *A. baumannii* 6772166 with AB0057 as the reference. The long red vertical lines on the bottom track represent the boundaries of each contig. A colored similarity plot is shown for each genome, the height of which is proportional to the level of sequence identity in that region.

Supplementary Figure 2 A global view of differential gene transcription induced by tigecycline. The circular map was based on the reference genome *A. baumannii* AB0057. From the outermost circle in, the circle 1 shows the ORFs on the plus strand of AB0057 chromosome, the circle 2 the ORFs on the minus strand, the circle 3 shows the degree of differential transcription (increased or decreased) for each gene, and the circle 4 shows the coordinates of the chromosome. The ORFs are color coded according to their respective COG category, and with the color scheme on the right. This map was constructed using a web-based tool Circular Visualization for Microbial Genomes (Overmars et al., 2015).

Supplementary Figure 3 Differential transcription of other membrane transporter encoding genes. Only the differently regulated membrane transporter genes are presented, and their absolute fold changes are more than 1 (log_2_ scale) with padj < 0.01. The genes on panels **(A)** and **(B)** are color grouped based on the putative substrate specificities. The genes on panel **(C)** are grouped based on transporter families, as they have no substrate predicted. Full information on gene function annotations is given in supplementary data.

Supplementary Figure 4 Increased transcription of transposase genes and DNA mobilization genes. For each functional group, only differentially transcribed genes with absolute fold changes >1 (log_2_ scale) with padj < 0.01 are shown. The bar charts are grouped by colors according to the biological functions of the genes assigned, with the color scheme on the right. Full information on gene function annotations is given in supplementary data.
