# Supplementary material for: The Transcriptomic Signature of Tigecycline in Acinetobacter baumannii
Source: Front Microbiol. 2020 Oct 27;11:565438. doi: 10.3389/fmicb.2020.565438 (PMC7652931; doi:10.3389/fmicb.2020.565438)
Supplement: Supplementary Figure 1 — Mauve visualization of genomic identity between A. baumannii AB0057 and A. baumannii 6772166. The horizontal track on the top represents the chromosome sequence of A. baumannii AB0057, and the one on the bottom represents the rearranged contig sequences of A. baumannii 6772166 with AB0057 as the reference. The long red vertical lines on the bottom track represent the boundaries of each contig. A colored similarity plot is shown for each genome, the height of which is proportional to the level of sequence identity in that region. [file Data_Sheet_1.docx]

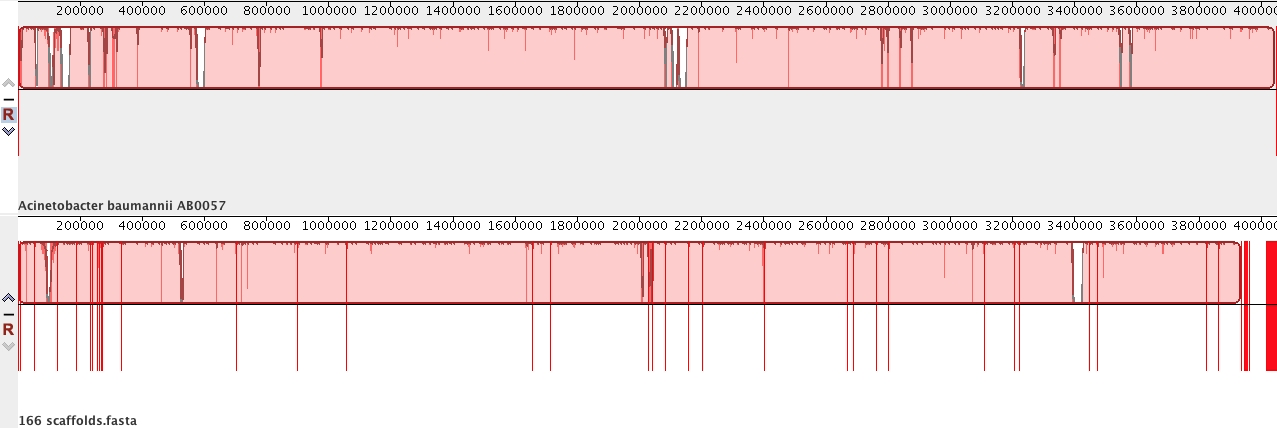


**Figure S1. Mauve visualization of genomic identity between *A. baumannii* AB0057 and *A. baumannii* 6772166.** The horizontal track on the top represents the chromosome sequence of *A. baumannii* AB0057, and the one on the bottom represents the rearranged contig sequences of *A. baumannii* 6772166 with AB0057 as the reference. The long red vertical lines on the bottom track represent the boundaries of each contig. A coloured similarity plot is shown for each genome, the height of which is proportional to the level of sequence identity in that region.


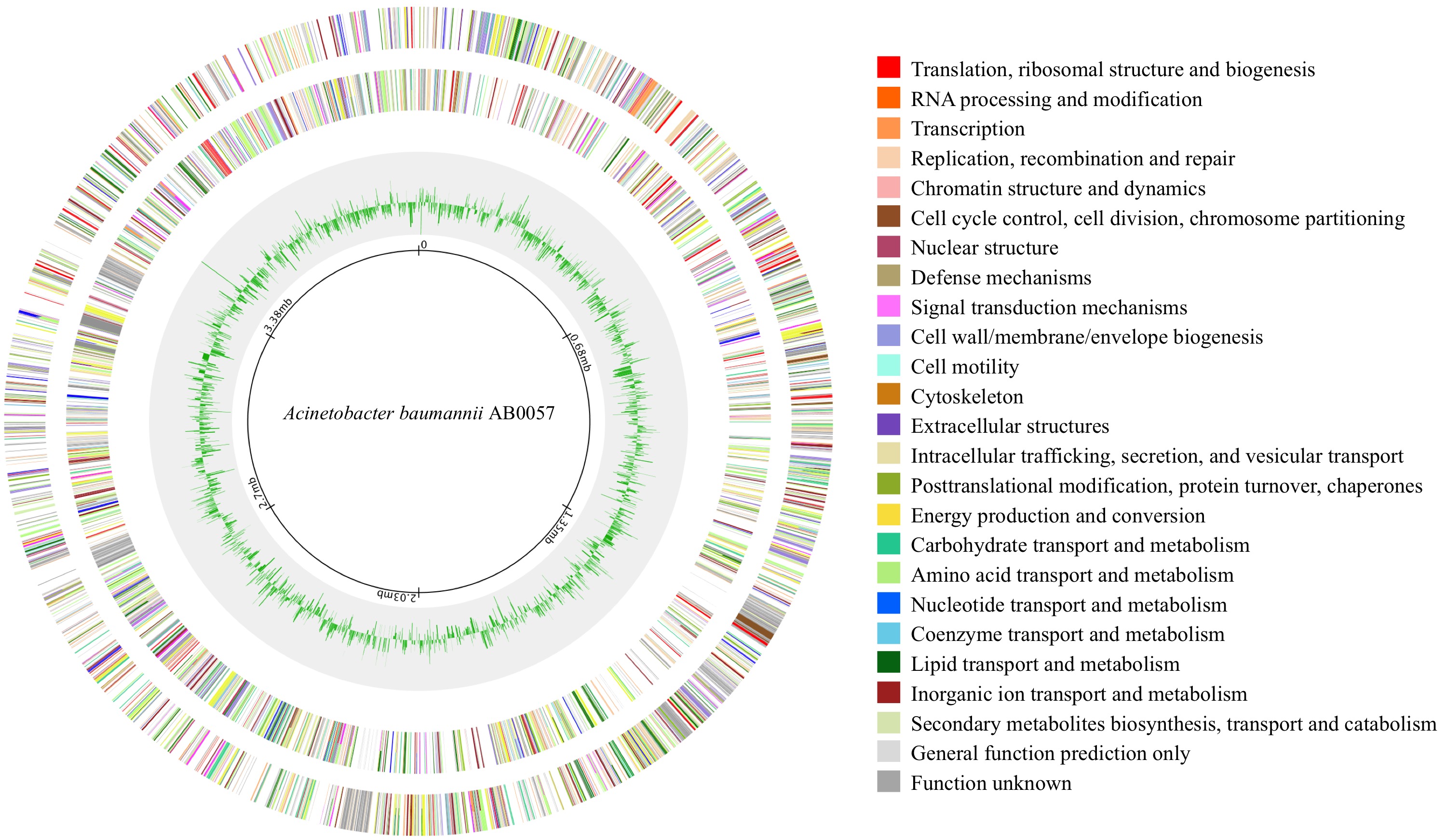


**Figure S2. A global view of differential gene transcription induced by tigecycline.** The circular map was based on the reference genome *A. baumannii* AB0057. From the outermost circle in, the circle 1 shows the ORFs on the plus strand of AB0057 chromosome, the circle 2 the ORFs on the minus strand, the circle 3 shows the degree of differential transcription (increased or decreased) for each gene, and the circle 4 shows the coordinates of the chromosome. The ORFs are colour coded according to their respective COG category, and with the colour scheme on the right. This map was constructed using a web-based tool Circular Visualization for Microbial Genomes (Overmars et al. 2015).


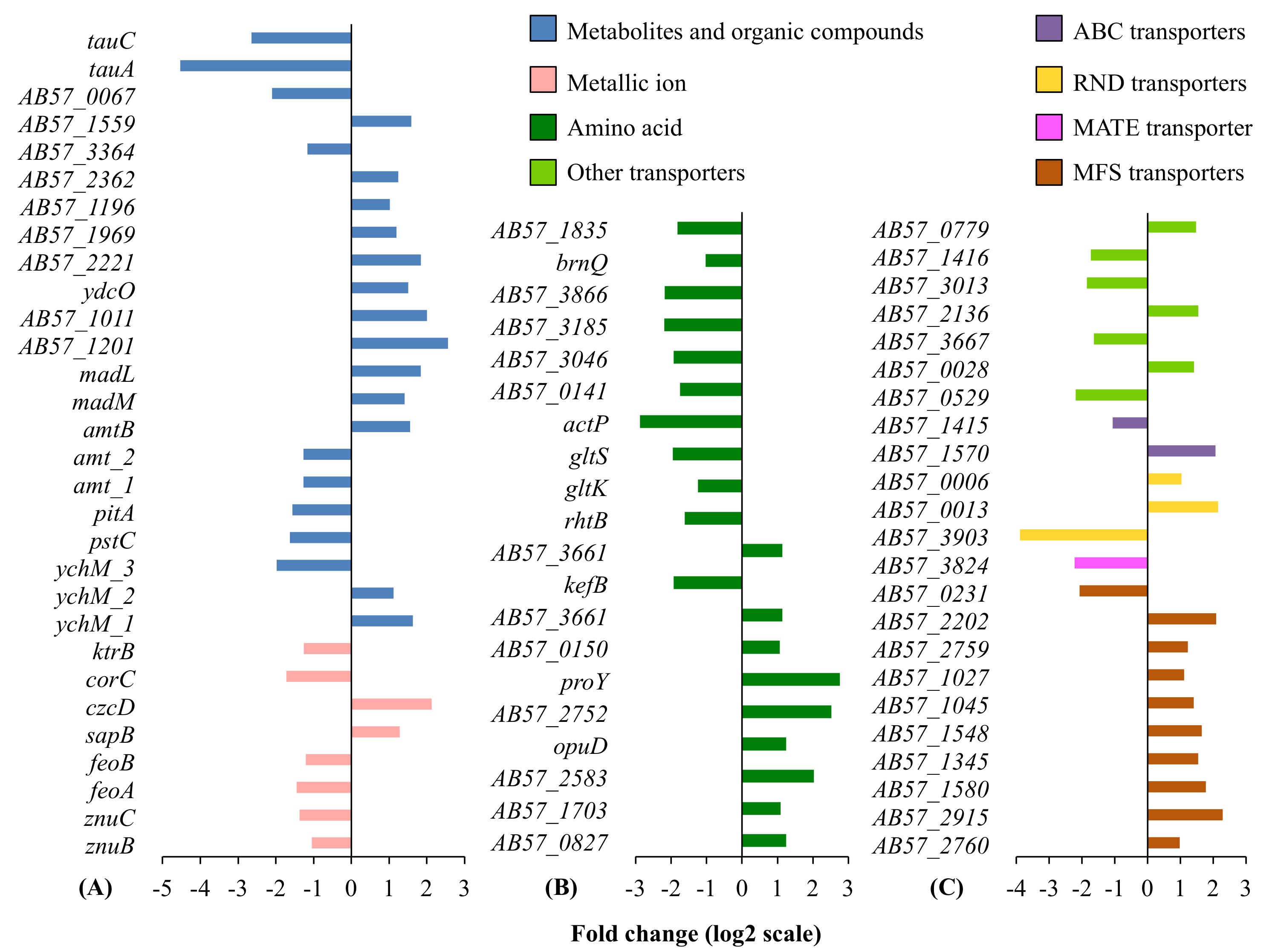


**Figure S3. Differential transcription of other membrane transporter encoding genes.** Only the differently regulated membrane transporter genes are presented, and their absolute fold changes are more than 1 (log_2_ scale) with padj < 0.01. The genes on panels A and B are colour grouped based on the putative substrate specificities. The genes on panel C are grouped based on transporter families, as they have no substrate predicted. Full information on gene function annotations is given in supplementary datasets.


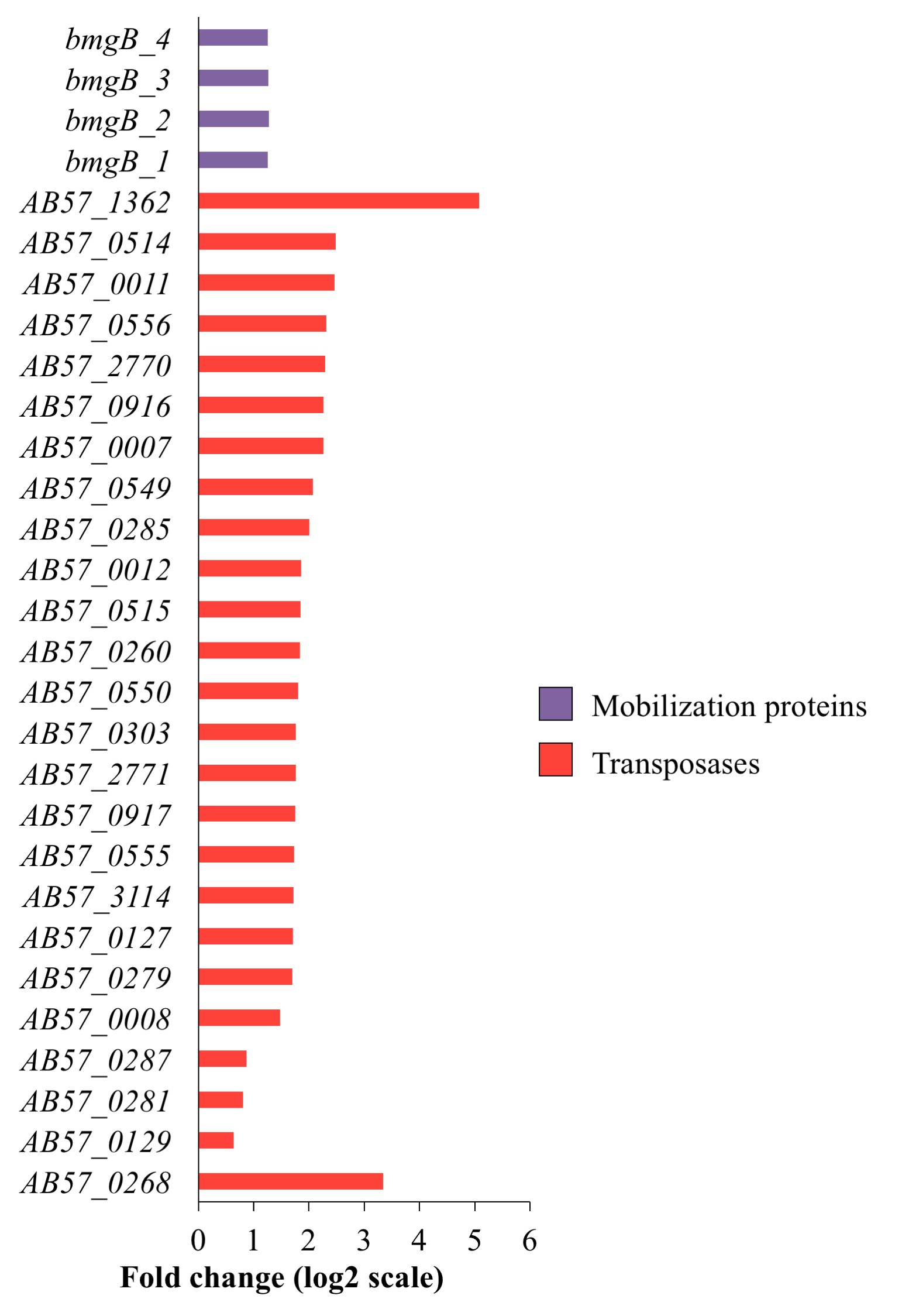


Figure S4. **Increased transcription of transposase genes and DNA mobilization genes.** For each functional group, only differentially transcribed genes with absolute fold changes > 1 (log_2_ scale) with padj < 0.01 are shown. The bar charts are grouped by colours according to the biological functions of the genes assigned, with the colour scheme on the right. Full information on gene function annotations is given in supplementary datasets.
